# Creation of a novel inverted charge density wave state

**DOI:** 10.1063/4.0000132

**Published:** 2022-01-13

**Authors:** Yingchao Zhang, Xun Shi, Mengxue Guan, Wenjing You, Yigui Zhong, Tika R. Kafle, Yaobo Huang, Hong Ding, Michael Bauer, Kai Rossnagel, Sheng Meng, Henry C. Kapteyn, Margaret M. Murnane

**Affiliations:** 1 Department of Physics and JILA, University of Colorado and NIST, Boulder, Colorado 80309, USA; 2 Beijing National Laboratory for Condensed Matter Physics and Institute of Physics, Chinese Academy of Sciences, Beijing 100190, China; 3 Shanghai Synchrotron Radiation Facility, Shanghai Advanced Research Institute, Chinese Academy of Sciences, Shanghai 201204, China; 4 Institute of Experimental and Applied Physics, Kiel University, D-24098 Kiel, Germany; 5 Ruprecht Haensel Laboratory, Deutsches Elektronen-Synchrotron DESY, 22607 Hamburg, Germany

## Abstract

Charge density wave (CDW) order is an emergent quantum phase that is characterized by periodic lattice distortion and charge density modulation, often present near superconducting transitions. Here, we uncover a novel inverted CDW state by using a femtosecond laser to coherently reverse the star-of-David lattice distortion in 1*T*-TaSe_2_. We track the signature of this novel CDW state using time- and angle-resolved photoemission spectroscopy and the time-dependent density functional theory to validate that it is associated with a unique lattice and charge arrangement never before realized. The dynamic electronic structure further reveals its novel properties that are characterized by an increased density of states near the Fermi level, high metallicity, and altered electron–phonon couplings. Our results demonstrate how ultrafast lasers can be used to create unique states in materials by manipulating charge-lattice orders and couplings.

## INTRODUCTION

The correlated interactions of electrons and atoms in crystalline materials, including electron–phonon coupling,^1,2^ play a crucial role in stabilizing emergent quantum phases such as charge density wave (CDW) order and superconductivity. Despite decades of research, the physics underlying these phases is still elusive. Recently, first-principle calculations of electron–phonon coupling have made it possible to predict the properties and behaviors of real materials.^1^ Techniques such as Raman,^3^ neutron,^4^ and x-ray scattering^5^ can probe phonon modes in the frequency domain and provide information about the integrated electron–phonon coupling over all electronic bands. For a comprehensive investigation of the electron–phonon coupling, one needs to determine the electron–phonon matrix element $g_{mn\nu }(k,q)$, which measures the coupling strength for the electron scattering from an initial state in band n with momentum k to a final state in band m with momentum k+q, via a phonon with mode ν at wave vector q. However, it remains challenging to experimentally extract momentum-resolved information about specific phonon modes interacting with particular electronic bands.

Ultrafast laser excitation of quantum materials can provide unique access to new light-induced states and their dominant couplings.^6–13^ When combined with advanced ultrafast spectroscopy techniques, it is now possible to study electron–phonon coupling in the time domain.^14–22^ In particular, time- and angle-resolved photoemission spectroscopy (trARPES) combines the direct measurement of the electronic structure with the ability to probe electron–phonon couplings on their intrinsic timescales—from femtoseconds on up. By monitoring either phonon-mediated electron scattering processes^23^ or band dynamics in the presence of coherent phonons,^24–27^ selectively coupled phonon modes can be investigated. Ultrafast laser-excited coherent phonons^28^ can modulate the electronic structure in a momentum-dependent way, which can be characterized by band- and mode-projected electron–phonon coupling strengths.^29,30^ Thus, how the electronic order responds to coherent phonons provides unique opportunities for studying and manipulating electron–phonon coupling in quantum materials and, importantly, maps the light-enriched phase diagram.

**FIG. 1.**
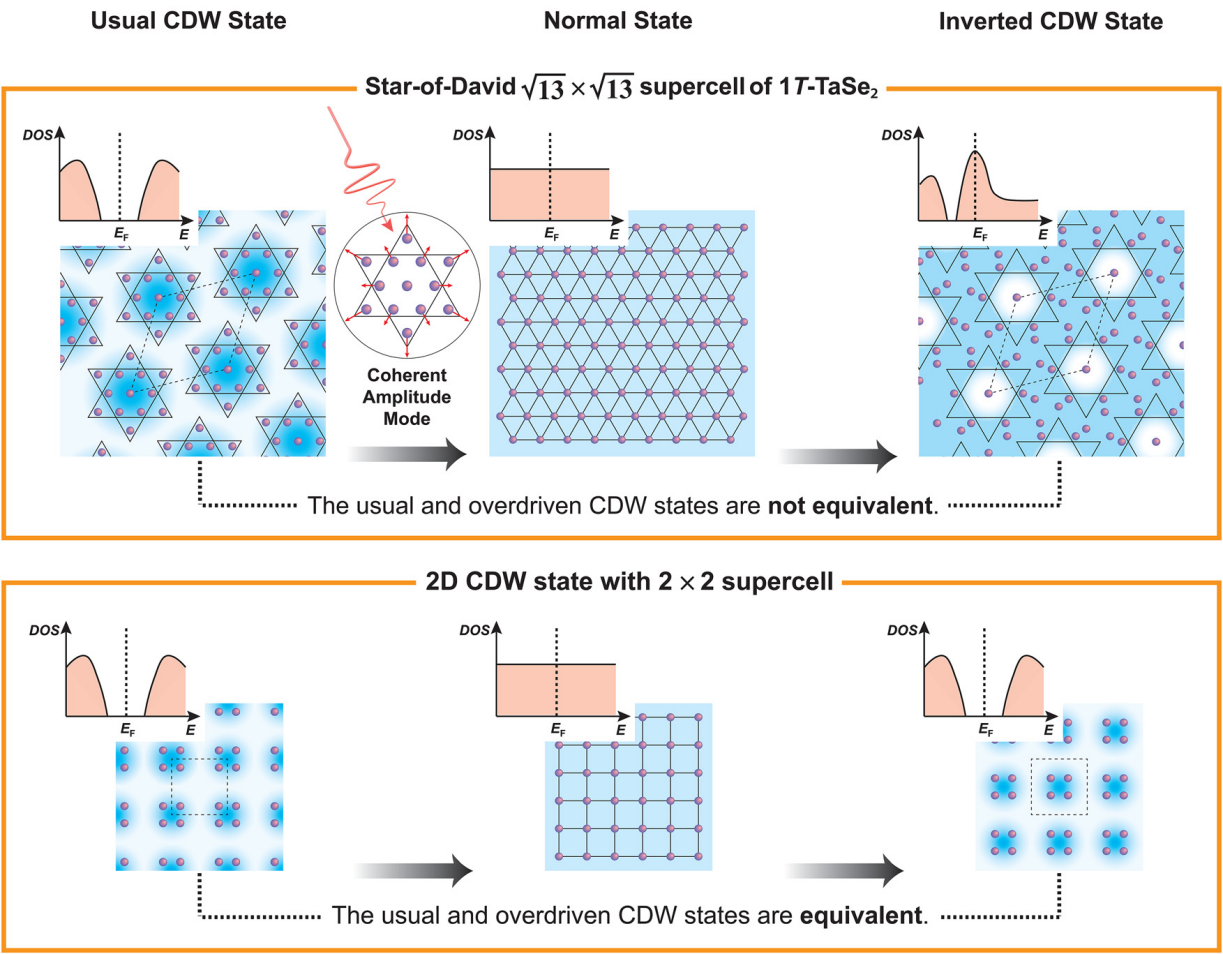
Generation of an inverted CDW state via ultrafast laser excitation of the coherent amplitude mode. The upper panel illustrates the evolution from the usual CDW state to the normal state and then to a new inverted CDW state in 1*T*-TaSe_2_. The blue shading represents the spatial electron density, while the purple circles represent in-plane Ta atoms with exaggerated lattice distortion. Because of the unique $\sqrt{13}\times \sqrt{13}$ supercell, the inverted CDW pattern is distinct from the usual one and shows high metallicity, as shown in the schematic density of states (the top inset of each panel). The lower panel shows an example case of a 2×2 supercell for comparison, where the usual and inverted CDW states are equivalent, considering the translational symmetry of the crystal.

In this work, we uncover a novel light-induced inverted CDW state in 1*T*-TaSe_2_ that has not been observed previously and validate its nature through a comparison of multiple experimental signatures with theory. This material has a unique star-of-David periodic lattice distortion that can be coherently excited by an ultrafast laser pulse. This launches a coherent CDW amplitude mode at ∼2 THz. Using trARPES, we can monitor the dynamic electronic structure and CDW order, which is encoded in the Ta 5*d* band position. For sufficiently strong laser excitation, the coherent amplitude mode drives the material from the usual CDW state, through the normal state, before entering a new inverted CDW state. In particular, this inverted CDW state is associated with an overshoot of the star-of-David periodic lattice distortion that supports a new CDW state exhibiting a high metallic character (Fig. 1). These results are confirmed by simulations based on the time-dependent density functional theory (TDDFT), which allow us to investigate band- and mode-projected electron–phonon coupling across different states.

**FIG. 2.**
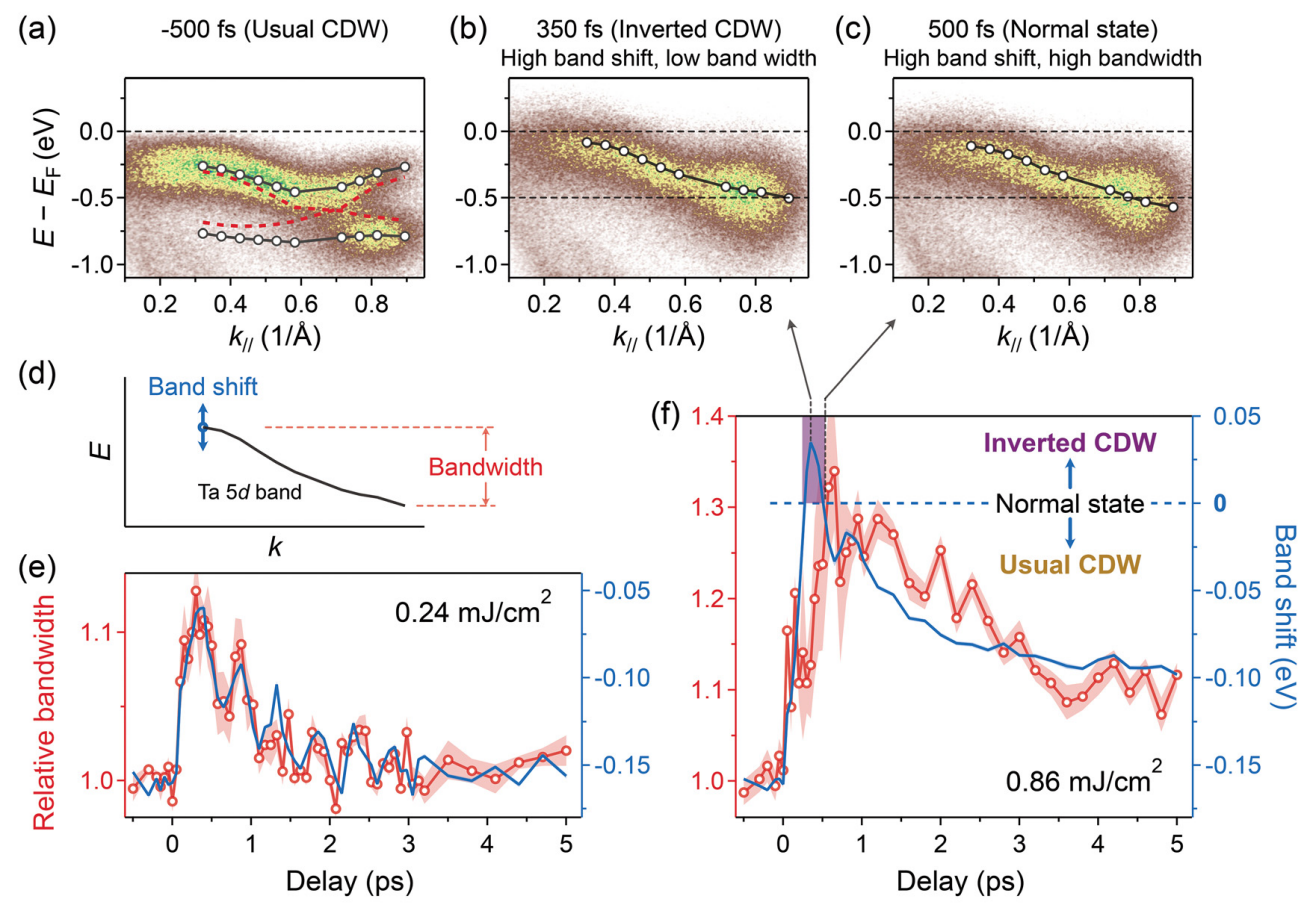
Experimentally observed Ta 5*d* band dynamics. (a) ARPES intensity plot along the Γ-M direction before laser excitation at usual CDW states. The black and red curves represent the band dispersions before and after removing the hybridization gap. (b) and (c) Same as (a) but for the inverted CDW and normal states after laser excitation, respectively. Despite the intensity broadening due to the limited energy resolution and high electron temperature, the band dispersions in (b) and (c) can be distinguished with the help of horizontal lines. (d) Definition of the band shift (energy shift at ∼0.3 Å^−1^ with respect to the normal state) and bandwidth (the energy range of the dispersion). (e) and (f) Temporal evolution of the band shift (blue) and the relative bandwidth compared to that before laser excitation (red) at fluences of 0.24 and 0.86 mJ/cm^2^, respectively. The shaded areas represent the error bars. Note that in (f) the horizontal blue dashed line aligned with a zero band shift corresponds to the normal state.

A great advantage of trARPES is that we can measure the band position, band dispersion, and band folding as well as the CDW gap, providing multiple observations to compare with theory. We find that the Ta 5*d* bandwidth [i.e., the energy range of the full dispersive band, see Fig. 2(d)] along the Γ–M direction increases as the material transforms from the usual CDW state into the normal state, before decreasing again as the material enters an inverted CDW state. In contrast, the band position shifts upwards monotonically. This evolution of bandwidth vs band position reveals a momentum-dependent electron–phonon coupling between the coherent amplitude mode and the Ta 5*d* band, and an inversion of deformation potential gradient in the momentum space when the material enters the new inverted CDW state—which is likely related to a change in the electron hopping interactions that are responsible for stabilizing emergent quantum phases.

## RESULTS

1*T*-TaSe_2_ is a typical two-dimensional (2D) CDW material. In the CDW state (below 470 K), the lattice reconstructs into a $\sqrt{13}\times \sqrt{13}$ star-of-David supercell, illustrated in the upper left panel of Fig. 1.^31^ The lattice reconstruction leads to band folding, giving rise to multiple band crossings and gap opening. Figure S1 shows the static ARPES measurements of the band structure at 300 K. In the Ta 5*d* band near the Fermi level (*E*_F_), two pronounced CDW gaps are observed along the Γ–M and M–K directions, respectively.

Our trARPES measurements employ a 1.6 eV pump pulse, followed by a 22.4 eV probe pulse produced from high harmonic generation (HHG). After laser excitation of 1*T*-TaSe_2_, the electrons thermalize to thousands of kelvins within ∼50 fs. The laser-excited electron redistribution smears out the charge localization very rapidly, which defines a new equilibrium position for the periodic lattice distortion and displacively launches the coherent amplitude mode.^11^ By strongly exciting this coherent mode, we can generate a unique and otherwise unreachable periodic lattice distortion (Fig. 1 top right)—that is very relevant to the emergent properties of 1*T*-TaSe_2_. This process can be dynamically monitored, because it is manifested by changes in multiple prominent features in the ARPES spectrum—including the Ta 5*d* band position, the CDW gap, and the band folding [Figs. 2(a)–2(c)]. The correlated dynamics of these features indicate that the band shift can represent the transient CDW order. The blue curves in Figs. 2(e) and 2(f) represent the time evolution of the band shift near the Γ point with respect to the normal state position [as illustrated in Fig. 2(d)] for two representative pump laser fluences. A systematic map that plots the band shift for a full range of laser fluences can be found in Fig. S2. The normal state (without any periodic lattice distortion) is indicated as the saturation level that the material is driven to as the excitation fluence increases.

When the laser excitation is sufficiently strong to completely and rapidly smear out the charge localization, the equilibrium point of the amplitude mode oscillation saturates at zero lattice distortion. However, as shown in Figs. 2(f) and S2, transient and collective overshooting of the periodic lattice distortion during its strong laser-driven oscillation (as monitored by the band shift) naturally leads to the formation of an inverted CDW state, which is characterized by a unique distortion, as illustrated in Fig. 1, top right. This novel inverted CDW state manifests within the first cycle of the amplitude mode that occurs between ∼250 and 500 fs after excitation. Thus, a high temporal resolution is required to detect this transient inverted CDW state, limiting the energy resolution to ∼130 meV. Although the detailed differences between the ARPES spectra of the inverted CDW and normal states are not readily visible by eye in the color maps in Figs. 2(b) and 2(c), the change in the band position and bandwidth can clearly be discerned with the help of the horizontal lines. Moreover, such differences become highly visible when the quantitatively extracted band shifts and widths are plotted over a wide range of time delays and laser fluences. Figures 2(f) and S2 present a clear signature of the overshoot feature that occurs between ∼250 and 500 fs after excitation, corresponding to a band shift in excess of the normal state. Interestingly, as the material is coherently excited from the usual CDW state to the normal state and then into the inverted CDW state, the Ta 5*d* band shifts up toward *E*_F_ monotonically, making this new state metallic. We note that as discussed in more detail in the supplementary material,^53^ the energy resolution is sufficient to resolve the ∼30 meV shift of the centroid of the single peak band shown in Fig. 2(f).

**FIG. 3.**
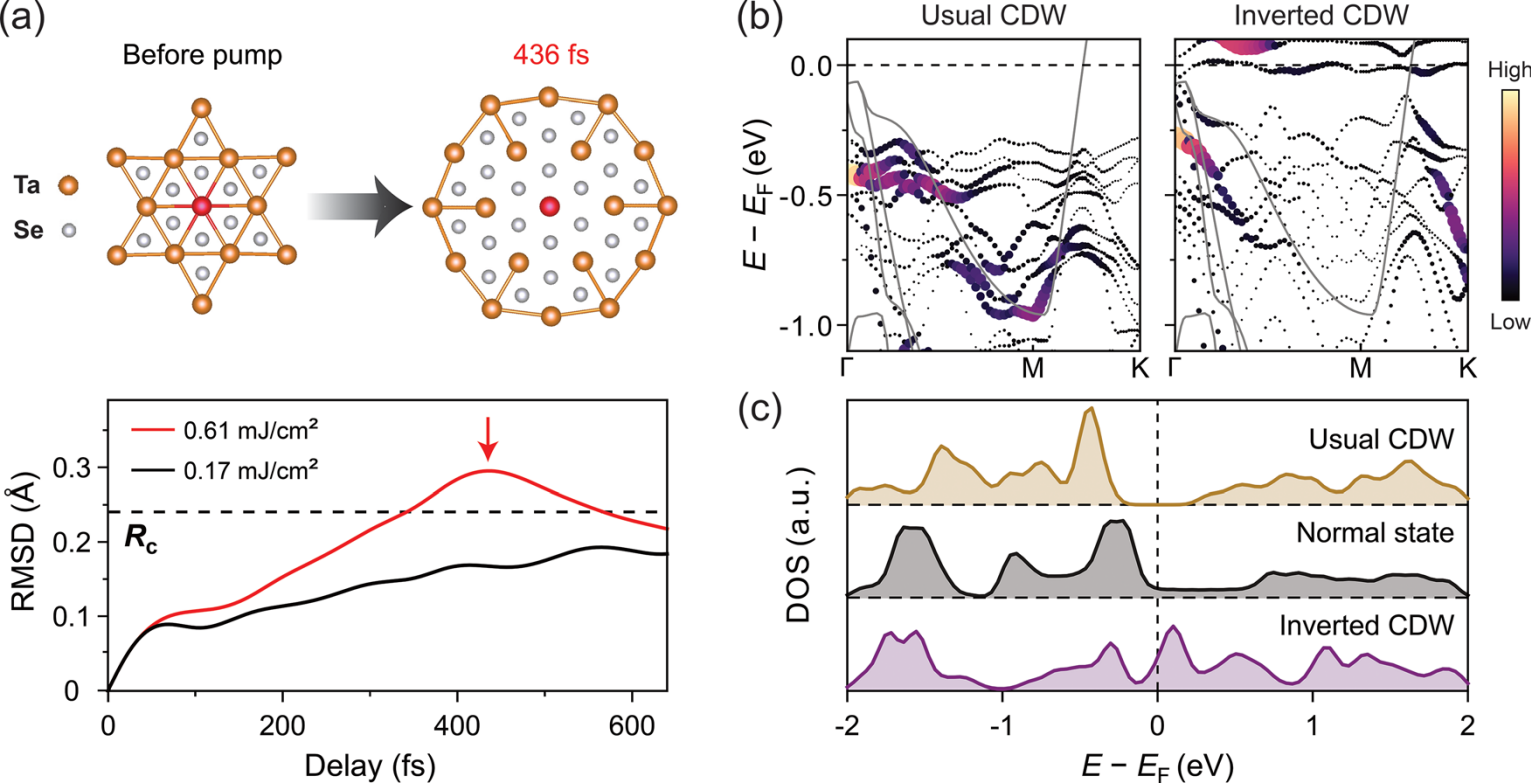
TDDFT simulations of the structural and electronic dynamics. (a) The lower panel shows the atomic root mean square displacement evolution for two representative laser fluences. The dashed line indicates the critical value *R*_c_ where the usual CDW order melts. The upper panel shows calculated structural patterns for the usual CDW and the inverted CDW (436 fs for a fluence of 0.61 mJ/cm^2^) states. In both plots, the bonds between the Ta–Ta atoms that are shorter than 3.5 Å are colored to show the usual CDW (left) and the inverted CDW order (right). (b) Calculated unfolded band structures for the usual and inverted CDW states, respectively, where the spectral weight is reflected by the brightness. Here the magnitude of the periodic lattice distortion of the inverted CDW is set to be the same as the usual CDW, that is beyond the experimental ones and thus we do not make a quantitative comparison. The unreconstructed band structure of the normal state is indicated by the gray line in each panel. (c) Calculated in-plane density of states corresponding to (b) for different states, the one of the inverted CDW state is the highest near *E*_F_.

To gain deep insight into the coherent electron and lattice dynamics after laser excitation, nonadiabatic molecular dynamic simulations based on TDDFT were carried out. The dynamics of the CDW phase under two laser fluences are shown in Fig. 3(a). To quantify the lattice structural changes, the root mean square displacement [RMSD $\left(t\right)$= $\sqrt{\mu^{2}\left(t\right)}]$ is calculated, where μ describes the atomic displacement of all atoms relative to the initial CDW structure. We adopt a criterion that the CDW state melts or transforms to a new phase when the RMSD reaches $R_{c}$ = 0.24 Å based on the fact that the CDW pattern is distorted by 7% from the normal state.^32^ For lower fluence (0.17 mJ/cm^2^), the RMSD increases slowly but stays below the melting threshold *R_c_*. However, for higher fluence (0.61 mJ/cm^2^), the RMSD value exceeds $R_{c}$ at 347 fs (close to the normal state) and reaches a maximum of 0.3 Å at 436 fs, accompanying the emergence of a new transient state. The calculated structure in the upper right panel of Fig. 3(a) confirms that it is indeed associated with an overshoot of the periodic lattice distortion (expanded star-of-David, rather than the contracted one that is accessed under equilibrium conditions). Importantly, the calculated unfolded band structures^33,34^ in Fig. 3(b) suggest that the inverted CDW state is highly metallic with larger density of states near *E*_F_ than the normal metallic state in strong contrast to the usual CDW state [Fig. 3(c)].

Note that the CDW patterns of the original and “inverted” CDW states are geometrically equivalent for many CDW materials such as K_0.3_MoO_3_,^35^ Cr,^36^ TiSe_2_,^37–39^ *R*Te_3_ (*R*: rare-earth element).^40^ As shown in the lower panel of Fig. 1 as an example, the sign change of the order (or a phase change of π) is not distinguishable, considering the translational symmetry of the crystal. In some cases, a domain wall between the original and inverted CDWs could be formed.^39^ Therefore, previously reported overshoots of CDWs in K_0.3_MoO_3_^35^ and Cr^36^ do not lead to a new structure. However, because of the special CDW wavevector associated with the $\sqrt{13}\times \sqrt{13}$ star-of-David supercell, the inverted CDW in 1*T*-TaSe_2_ is significantly distinct from the usual CDW in terms of the lattice order (upper panel of Fig. 1), as well as the electronic structure, which defines it as a truly new state. For this reason, this inverted CDW is energetically unfavorable and, thus, challenging (if not impossible) to reach under normal conditions. Interestingly, the distinction between the star-of-David and inverse star-of-David structures is also being discussed in the recently discovered kagome superconductors *A*V_3_Sb_5_ (*A*: K, Rb, Cs), and the inverse star-of-David, which can be stabilized under thermal equilibrium in the kagome lattice, was even proposed to be the CDW ground state.^41–43^

**FIG. 4.**
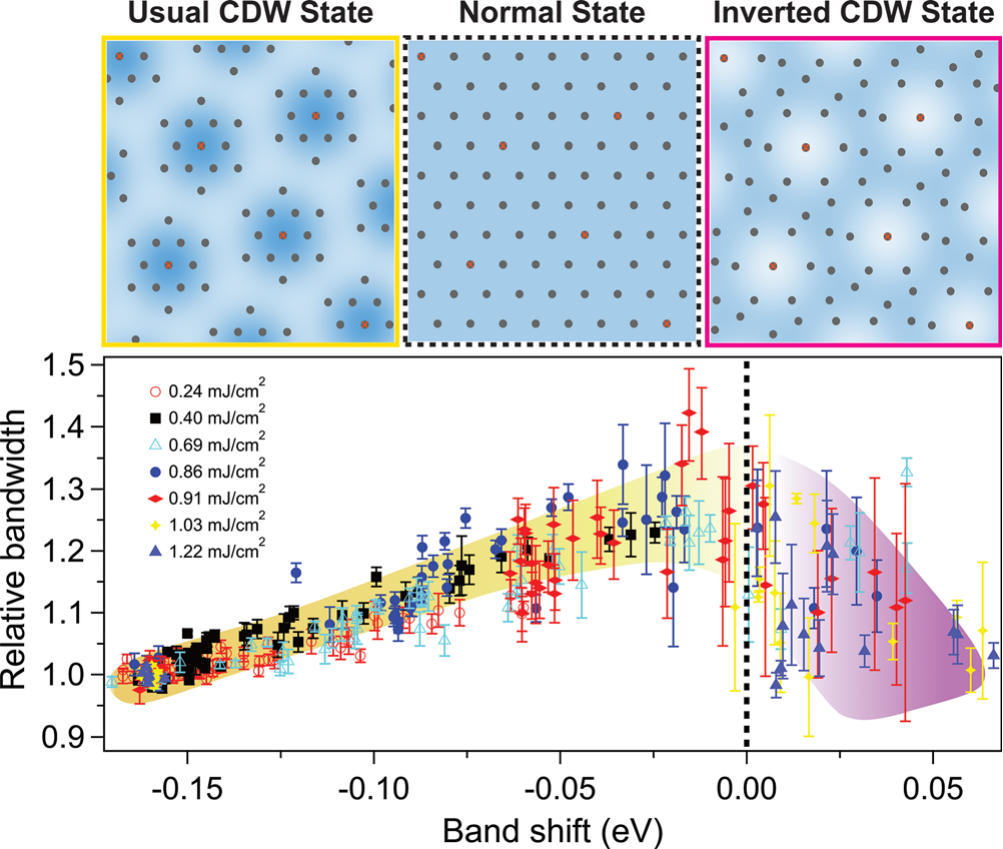
The bandwidth as a function of the band shift over a wide range of fluences and time delays. This universal relation, regardless of fluence and time delay, can classify different states with the lattice structures illustrated in upper panels. The yellow and purple shadows are the guide to the eye in the usual and inverted CDW states, respectively. Although the error bars are relatively large on the inverted CDW side because of the spectrum broadening in such highly excited states, the reversal of the trend is still clearly observed.

To further investigate the properties of this novel inverted CDW state, we monitor the Ta 5*d* bandwidth [as defined in Fig. 2(d)], which is closely related to the electron–electron correlation and/or the electron–phonon coupling.^1,44–47^ Note that the bandwidth is also strongly modified by the band folding and hybridization, and we take this into account. In Fig. 2(a), the two original unreconstructed bands (red curves) can be extracted from the ARPES spectra after removing the hybridization gap using a mean-field approximation (see the supplementary material^53^). Figures 2(e) and 2(f) plot the dynamics of this bandwidth together with the band shift for two typical laser pump fluences. For lower fluence (0.24 mJ/cm^2^), the bandwidth and band shift dynamics are similar, both exhibiting coherent oscillations. However, for higher fluence (0.86 mJ/cm^2^), they deviate from each other at early time delays. Figure 4 plots the bandwidth as a function of the band shift over a wide range of fluence, which shows a universal relation and clearly highlights the different regimes. The bandwidth increases as the material evolves from the usual CDW state to the normal state. However, this trend reverses as the material enters the inverted CDW state. Although this result is not required to support the presence of an inverted CDW state, it is physically reasonable considering the differences in the lattice orders, as also confirmed by the DFT calculations [Fig. 3(b)].

## DISCUSSION

**FIG. 5.**
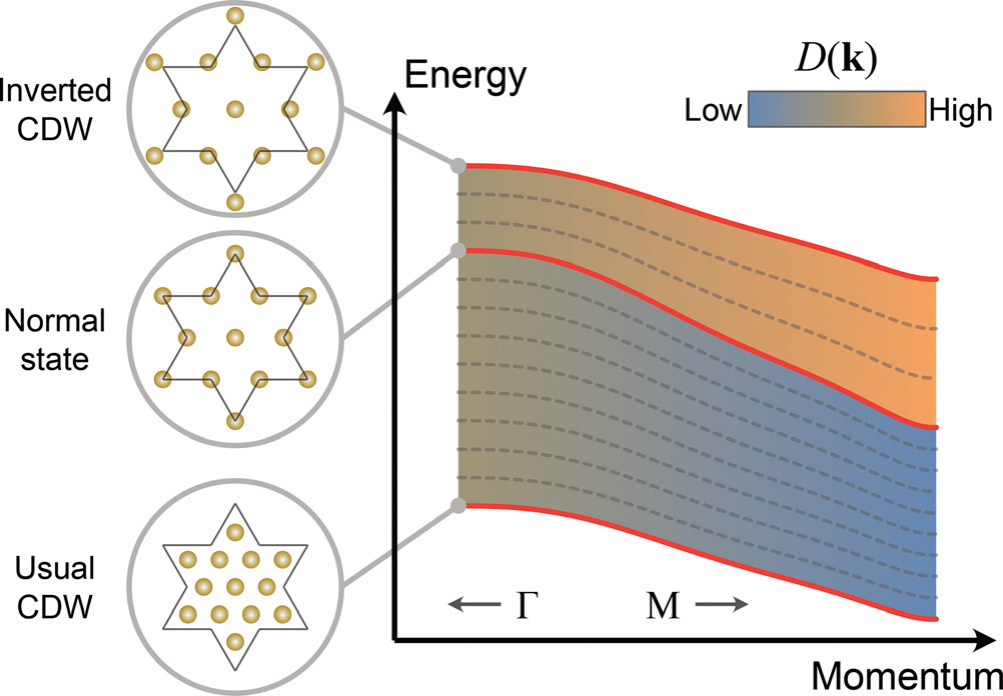
Qualitative schematic of the momentum-dependent electron–phonon coupling in different states. The red solid lines and gray dashed lines represent the Ta 5*d* band dispersions corresponding to different periodic lattice distortions. The color scale of the shading represents the qualitative momentum dependence of the deformation potential D(k). Between the usual CDW state and the normal state, the deformation potential at the Γ point is larger than that at the Μ point, while such gradient is reversed between the normal state and the inverted CDW state.

Such a relation between the bandwidth and the band shift shown in Fig. 4 provides unique insight into mode-projected electron–phonon coupling during the ultrafast phase transitions. The momentum-dependent band shift of the Ta 5*d* band, which leads to a dynamical bandwidth, can be characterized by the momentum-dependent deformation potential for the specific amplitude mode: $D\left(k\right)=\delta \varepsilon (k)/\delta u$, where δε(k) is the band shift and δu is the variation of the phonon coordinate. D(k), which determines the electron–phonon coupling strength, plays a pivotal role in structural phase transitions. The band dispersions across the three material states we probe are schematically presented in Fig. 5. The color scale of the shading qualitatively represents the evolution of D(k) in the momentum space. Between the usual CDW and normal states, D(k) at the Γ point is larger than that at the Μ point, while the trend is reversed on the inverted CDW side. When the periodic lattice distortion crosses zero, there is a change in both the sign and magnitude of $\partial D\left(k\right)/\partial k$, as manifested by the opposite and asymmetric slope (bandwidth vs band shift) in Fig. 4. This inversion of deformation potential gradient could be related to the unique expanded David-stars in the inverted CDW state compared to the usual contracted ones (Fig. 1). Such a lattice rearrangement significantly modifies the inter-star, intra-star, and interlayer electron hopping interactions, which result in a narrowing of the bandwidth in the inverted CDW state. Future research such as ultrafast diffraction experiments could provide deeper understandings on this fundamental problem.

In summary, we demonstrate the creation of a novel inverted CDW state in the 2D CDW material 1*T*-TaSe_2_ using trARPES and TDDFT. An intense femtosecond laser pulse can strongly excite the coherent phonon to drive the material into an otherwise unreachable state with unique order. Time-resolved ARPES can then monitor the dynamic electronic response to this specific mode in a band- and momentum-resolved manner. This allows us to investigate the electronic structure and electron–phonon couplings in many unique lattice configurations and further stimulate the advanced theory.

## MATERIALS AND METHODS

### Experiments

Time-resolved ARPES spectra were collected with a home-built setup using 22.4 eV extreme ultraviolet (EUV) photons and the SPEC PHOIBOS 100 analyzer. The 1.6 eV infrared laser beam with ∼30 fs pulse duration and 4 kHz repetition rate (KMLabs Dragon) was tightly focused into a hollow fiber filled with Kr gas to generate EUV photons. The single crystal samples were cleaved and measured at room temperature under a vacuum of $3\times 10^{-10}$ Torr. The overall energy resolution of about 130 meV is mainly limited by the spectral width of the ultrashort EUV pulses. The pump laser fluence was limited in a range to avoid discernible space-charge effects on the ARPES spectra. Static ARPES measurements were performed at the “Dreamline” beamline at Shanghai Synchrotron Radiation Facility with a Scienta Omicron DA30L analyzer. The overall energy resolution was about 40 meV. The samples were also cleaved and measured at room temperature under a vacuum better than $1\times 10^{-10}$ Torr.

### Theoretical calculations

The nonadiabatic dynamics calculations were performed using the time-dependent *ab initio* package (TDAP) as implemented in SIESTA.^48–50^ The bulk 1*T*-TaSe_2_ in its usual CDW state was simulated with a supercell of 78 atoms with $\sqrt{13}\times \sqrt{13}\times 2$ periodical boundary conditions. Numerical atomic orbitals with double zeta polarization (DZP) were employed as the basis set. The electron–nuclear interactions were described by Troullier–Martins pseudopotentials and the Perdew–Burke–Ernzerhof (PBE) functional.^51^ An auxiliary real-space grid equivalent to a plane wave cutoff of 250 Ry was adopted. The coupling between atomic and electronic motions was governed by the Ehrenfest approximation.^52^ During dynamic simulations, the evolving time step was set to be 0.05 fs for both electrons and ions in a micro-canonical ensemble. Linearly polarized laser beams with time-dependent electric field $E\left(t\right)=E_{0}\text{cos}(\omega t)\exp\left[-{\left(t-t_{0}\right)}^{2}/2\sigma^{2}\right]$ were applied to the usual CDW ground state of 1*T*-TaSe_2_. The photon energy ℏω and pulse duration were set as 1.6 eV and 14 fs, respectively.

## Data Availability

The data that support the findings of this study are available within the article and its supplementary material..
